# Modelling and visualizing fine-scale linkage disequilibrium structure

**DOI:** 10.1186/1471-2105-14-179

**Published:** 2013-06-06

**Authors:** David Edwards

**Affiliations:** 1 Department of Molecular Biology and Genetics, Centre for Quantitative Genetics and Genomics, Blichers Allé 20, Tjele 8830, Denmark

## Abstract

**Background:**

Detailed study of genetic variation at the population level in humans and other species is now possible due to the availability of large sets of single nucleotide polymorphism data. Alleles at two or more loci are said to be in linkage disequilibrium (LD) when they are correlated or statistically dependent. Current efforts to understand the genetic basis of complex phenotypes are based on the existence of such associations, making study of the extent and distribution of linkage disequilibrium central to this endeavour. The objective of this paper is to develop methods to study fine-scale patterns of allelic association using probabilistic graphical models.

**Results:**

An efficient, linear-time forward-backward algorithm is developed to estimate chromosome-wide LD models by optimizing a penalized likelihood criterion, and a convenient way to display these models is described. To illustrate the methods they are applied to data obtained by genotyping 8341 pigs. It is found that roughly 20% of the porcine genome exhibits complex LD patterns, forming islands of relatively high genetic diversity.

**Conclusions:**

The proposed algorithm is efficient and makes it feasible to estimate and visualize chromosome-wide LD models on a routine basis.

## Background

Alleles at two loci are said to be in linkage disequilibrium (LD) when they are correlated or statistically dependent. The term refers to the idea that in a large homogeneous population subject to random mating, recombination between two loci will cause any initial association between them to vanish over time. In observed data, however, non-zero allelic associations are pervasive, particularly at short distances, but also at long distances and even between chromosomes. These associations arise in a complex interplay between processes such as mutation, selection, genetic drift and population admixture, and are broken down by recombination. The patterns of association are of interest, partly because they underpin the relation of genotype to phenotype at the population level, and partly because they reflect population history.

Patterns of LD may be represented in different ways. A common method is to display pairwise measures of LD as triangular heatmaps [1,2]: in these displays, LD blocks (genomic intervals within which all loci are in high LD) stand out clearly. Early work in the HapMap project led researchers to hypothesize that the human genome consists of a series of disjoint blocks, within which there is high LD, low haplotype diversity and little recombination, and that are punctuated by short regions with high recombination (recombination hotspots) [3-6]. Subsequently various authors [7,8] reported that genetic variation follows more complex patterns, for which richer models are required.

Discrete graphical models [9] (also known as discrete Markov networks) provide a rich family of statistical models to describe the distribution of multivariate discrete data. They may be represented as undirected graphs in which the nodes represent variables (here, SNPs) and absent edges represent conditional independence relations, in the sense that two variables that are not connected by an edge are conditionally independent given some other variables. To motivate this focus on conditional rather than marginal associations, consider three loci $s_{1},\ldots s_{3}$, and suppose that initially *s*_2_ is polymorphic and *s*_1_ and *s*_2_ monomorphic, so that two haplotypes (1,1,1) and (1,2,1) are initially present. Suppose further that a mutation subsequently occurs at *s*_1_ in the haplotype (1,1,1), and another at *s*_3_ in the haplotype (1,2,1), so that the population now contains the four haplotypes (1,1,1), (1,2,1), (2,1,1) and (1,2,2). Observe that in general *s*_1_ and *s*_3_ are marginally associated (are in LD), but in the subpopulations corresponding to *s*_2_=1 and *s*_2_=2 they are unassociated: in other words, they are conditionally independent given *s*_2_. More complex mutation histories give rise to more complex patterns of conditional independences that can be represented as graphical models [8].

Other authors have used graphical models for the joint distributions of allele frequencies. Usually, in high-dimensional applications, attention is restricted to a tractable subclass, the *decomposable* graphical models [10]. In the first use of decomposable models in this context [11], models were selected using a greedy algorithm based on significance tests. In [8,12] methods and programs for selecting decomposable graphical models using Monte Carlo Markov Chain (MCMC) sampling were described. These methods are computationally feasible for modest numbers of markers (say, several hundreds), but not for modern SNP arrays with hundreds of thousands of SNPs per chromosome. To improve efficiency, the search space may be restricted to graphical models whose dependence graphs are *interval graphs*[13,14]. These are graphs for which each vertex may be associated with an interval of the real line such that two vertices are connected by an edge if and only if their intervals overlap. In this context the ordering of SNPs along the real line is their physical ordering along the chromosome. MCMC sampling from this model class may be performed more efficiently [13,14]. This work was extended in [15] to a more general subclass of decomposable models, namely those in which distant marker pairs (i.e., with more than a given number of intervening markers) are conditionally independent given the intervening markers.

In an alternative approach [16-18] latent mixtures of forests have been applied, in order to accommodate short-, medium- and long-range LD patterns. Also directed graphs (Bayesian networks) have been applied, selecting edges and their directions using causal discovery algorithms [19]. There are close links between decomposable models and Bayesian networks ([10], Sect. 4.5.1).

A rather different approach to modelling the joint distribution of allele frequencies [20,21] is implemented in the software package BEAGLE [22], which is widely used to process data from SNP arrays. The approach is based on a class of models arising in the machine learning literature called *acyclic probabilistic finite automata* (APFA) [23]. These are related to time-variant variable length Markov chains. For phase estimation and imputation BEAGLE uses an iterative scheme analogous to the EM algorithm, alternating between sampling from a haplotype-level model given the observed genotype data (the E-step) and selecting a haplotype-level model given the samples (the M-step). A similar computational scheme for decomposable graphical models has been described and implemented in the FitGMLD program [15].

Characterization of genetic variation at the population level is of fundamental importance to understanding how phenotypes relate to genotypes. Some specific uses to which joint models for allele frequencies have been put include 

1. Insight into the population history of different genomic intervals. Under simplifying assumptions, the ancestral history of a short genomic interval can be reconstructed from a decomposable graphical model for the SNPs in the interval [8].

2. Quality control of genome assembly, in that some motifs may suggest errors in SNP positioning.

3. Phase estimation and imputation as described above [15,21].

4. Derivation of more informative covariates involving multiple loci to plug into genomic prediction models [20].

5. Use in pedigree simulation to model LD in founders, for example in connection with gene drop simulation [14] and assessment of SNP streak statistics [24].

In this paper decomposable graphical models are used to model fine-scale, local LD patterns. By *local* is meant that the proposed methods are designed to capture short-range associations between loci, but not long range ones. The same is true of other approaches [13-15] mentioned above. Here, an efficient, linear-time algorithm is developed to select a model using a penalized likelihood criterion, and it is shown how such a model may conveniently be displayed, allowing fine-scale LD structure to be visualized.

## Methods

### Graphs and graphical models

The following notation and terminology is mainly based on [9]. A graph is defined as a pair G=(V,E), where *V* is a set of *vertices* or *nodes* and *E* is a set of *edges*. Each edge is associated with a pair of nodes, its *endpoints*. Here only undirected graphs are considered, that is, with graphs undirected edges only. Two vertices *α* and *β* are said to be *adjacent*, written *α*∼*β*, if there is an edge between them. The *neighbours* of a vertex is the set of nodes that are adjacent to it. A subset *A*⊆*V* is *complete* if all vertex pairs in *A* are connected by an edge. A *clique* is a maximal complete subset, that is to say, a complete subset that is not contained in a larger complete subset.

A *path* (of length *n*) between vertices *α* and *β* in an undirected graph is a set of vertices $\alpha =\alpha_{0},\alpha_{1},\ldots ,\alpha_{n}=\beta$ where *α*_*i*−1_∼*α*_*i*_ for i=1,…,n. If a path $\alpha =\alpha_{0},\alpha_{1},\ldots ,\alpha_{n}=\beta$ has *α*=*β* then the path is said to be a *cycle* of length *n*. If a cycle $\alpha =\alpha_{0},\alpha_{1},\ldots ,\alpha_{n}=\alpha$ has adjacent elements *α*_*i*_∼*α*_*j*_ with *j*∉{*i*−1,*i*+1} then it is said to have a *chord*. If it has no chords it is said to be *chordless*. A graph with no chordless cycles of length ≥4 is called *triangulated* or *chordal*.

A subset *D*⊂*V* in an undirected graph is said to *separateA*⊂*V* from *B*⊂*V* if every path between a vertex in *A* and a vertex in *B* contains a vertex from *D*. The graph $G_{0}=(V_{0},E_{0})$ is said to be a *subgraph* of G=(V,E) if *V*_0_⊆*V* and *E*_0_⊆*E*. For *A*⊆*V*, let *E*_*A*_ denote the set of edges in *E* between vertices in *A*. Then $G_{A}=(A,E_{A})$ is the *subgraph induced byA*.

The *boundary* bd(*A*) of a vertex set *A*⊆*V* is the set of vertices adjacent to a vertex in *A* but not in A, that is ,.

bd(A)={v∈V:v∼wfor somew∈A}∖A.

Let G=(V,E) be an undirected graph with cliques $C_{1},\ldots C_{k}$. Consider a joint density *f*() of the variables in *V*. If this admits a factorization of the form

$$f(x_{V})=\overset{k}{\underset{i=1}{\prod }}g_{i}(x_{C_{i}})$$

for some functions $g_{1}()\ldots g_{k}()$ where *g*_*j*_() depends on *x* only through $x_{C_{j}}$ then *f*() is said to factorize according to G. If all the densities in a model factorize according to G, then the model is said to be G-Markov. When this is true G encodes the conditional independence structure of the model, through the following result (the *global Markov property*): whenever sets *A* and *B* are separated by a set *C* in G, *A* and *B* are conditionally independent given *C* under the model. This is written as *A*⊥ ⊥*B* | *C*. A *decomposable* graphical model is one whose dependence graph is triangulated.

### Selecting graphical models for chromosome-wide LD

Suppose that *N* observations of *p* SNPs from the same chromosome are sampled from some population. The variable set is written $V=(v_{1},\ldots v_{p})$, and it is assumed that these are ordered by physical position on the chromosome. The variables may be either observed genotypes or inferred haplotypes, if these have been imputed: the former are trichotomous and the latter binary. Here an algorithm to use these data to select a graphical model for the distribution of *V* is described. It is based on a penalized likelihood criterion

(1) $$\text{IC}(G)=-2{\hat{\ell }}_{G}+\alpha \;\dim(G)$$

where ${\hat{\ell }}_{G}$ is the maximized log-likelihood under G, dim(G) is the number of free parameters under G, and *α* is a penalization constant. For the AIC, *α*=2 and for the BIC, *α*= log(*N*). The latter penalizes complex models more heavily and so selects simpler models. Under suitable regularity conditions, the BIC is consistent in the sense that for large *N* it will select the simplest model consistent with the data ([25], Sect. 2.6).

A technical but nonetheless important point is that calculation of the correct model dimension is not straightforward for high-dimensional models, since not all parameters may be estimable. A simple expression exists however for the difference in model dimension between two decomposable models that differ by a single edge ([10], pp. 37-40). This is useful when the search space is restricted to decomposable models and the search algorithm only involves comparison of models differing by single edges (as here).

A forward-backward approach to estimate the graphical model for *V* is used. The first (forward) step is based on a greedy forward search algorithm. To take account of the physical ordering of the SNPs, the algorithm starts from a model $G_{0}=(V,E_{0})$ where *E*_0_ is the set of edges between physically adjacent SNPs: this model is called the *skeleton*. To seek the minimum BIC (or AIC) model, the algorithm repeatedly adds the edge associated with the greatest reduction in BIC (AIC): only edges whose inclusion results in a decomposable model are considered. The process continues until no more edges improve the criterion. The search space in this step is the class of decomposable models that include the skeleton. Note that this algorithm – as with almost all greedy algorithms — is not guaranteed to find the global optimum.

There are several advantages to initially constraining the search space to include the skeleton. An unconstrained search starting off from the null model (with no edges) would not take the physical ordering into account. Since the graphs considered are consistent with this ordering, they are conveniently displayed as LD maps, as illustrated below. Because decomposable models contain no chordless cycles of length ≥4, two distal loci cannot be linked by an edge unless there are sufficiently many intermediate (chordal) edges to prevent the occurrence of such cycles. Thus the algorithm proceeds by filling out around the skeleton by incrementally adding edges. The effect of this is that only local LD is modelled. Since both association and (implicitly) proximity are required, the edges included are more likely to be for real (that is, less likely to be due to chance), so in this sense the model selection process is more reliable. In addition, restricting the search space in this way improves computational efficiency. The linear-time algorithm described in the following section also builds on the physical vertex ordering.

In the second (backward) step the graph found in the first step is pruned, again using a greedy algorithm that seeks to minimize the BIC (AIC) criterion by removing edges, without requiring that the skeletal edges are retained. Keeping these in the model would be appropriate if adjacent SNPs are in high LD, but this is not always the case. For example, there may be recombination hotspots, or genome assembly errors may have led to errors in positioning the SNPs. The graphs may be used to examine whether this has occurred.

To display the resulting graph, a suitable graph layout is needed. After some experimentation, one such was obtained by modifying the horizontal dot layout in Rgraphviz [26], by exponentially smoothing the *y*-coordinates and replacing the *x*-coordinates with the SNP-number. In this way the *y*-coordinates are chosen so as to clarify the graphical structure: a consequence of this is that nodes with large absolute *y*-coordinates tend to signal genomic regions of high structural complexity.

For some purposes it is helpful to use more objective measures of complexity, and two such measures are used here. Consider a chromosome with *p* SNPs. For each i=1,…p−1, the *heighth*_*i*_ of the interval between SNPs *i* and *i*+1 is defined to be the number of edges of the form (*j*,*k*), where *j*≤*i* and *i*+1≤*k*, and the *widthw*_*i*_ is defined to be the maximum value of |*k*−*j*| of such edges. Note that by the global Markov property, when *h*_*i*_=0 or equivalently *w*_*i*_=0, the SNPs *V*_≤*i*_={*v*_*j*_:*j*≤*i*} are independent of *V*_>*i*_={*v*_*j*_:*j*>*i*}. Similarly, when *h*_*i*_=1 or equivalently *w*_*i*_=1, *V*_<*i*_⊥⊥*V*_>*i*_|*v*_*i*_.

As a measure of haplotype diversity, the *entropy*[27] is used here. Suppose that in some genomic interval there are *k* distinct haplotypes with relative frequencies $f_{1},\ldots f_{k}$. Then the entropy is defined as $H=-\sum_{i=1\ldots k}f_{i}\log f_{i}$. It is large when there are many distinct haplotypes that are equally frequent.

A useful way to display marker data over short genomic intervals is the *sample tree*[23]. This summarizes a set of discrete longitudinal data of the form $x^{\left(v\right)}=(x_{1}^{\left(v\right)},\ldots x_{q}^{\left(v\right)})$ for v=1…N. A rooted tree is constructed in which the non-root nodes represent partial outcomes of the form $\left(x_{1},\ldots x_{k}\right)$ for *k*≤*q* present in the sample data. Edges may be coloured with the marker value and labelled with the corresponding sample counts, or drawn with width proportional to the sample counts.

### A fast selection algorithm

For high-dimensional models, the first step of the algorithm described above can be computationally demanding. To address this a much faster algorithm for the same task is now described. This involves combining a series of overlapping marginal models of lower dimension. Call the block length (model dimension) *L* and the overlap *K*. Thus the first block is $V_{1}=\{v_{1},\ldots v_{L}\}$, the second is $V_{2}=\{v_{L-K+1}\ldots v_{2L-K}\}$ and so on. In the applications described here *L*=100 and *K*=20 are used.

Suppose that the true model for $V=(v_{1},\ldots v_{p})$ is G, and that $\hat{G}$ is the estimate of G obtained by performing the first step of the algorithm described in the previous section. The goal is to construct an approximation $\tilde{G}$ to $\hat{G}$ by combining models ${\hat{G}}_{i}=(V_{i},{Ê}_{i})$ obtained by applying that same algorithm to blocks *V*_*i*_ for i=1,2….

A way to construct a model ${\tilde{G}}_{12}$ for *V*_1_∪*V*_2_ by combining ${\hat{G}}_{1}$ and ${\hat{G}}_{2}$ is now described. Let $m^{*}=\max\{w:\exists \;(v,w)\in {Ê}_{1}\;\text{with}\;v\leq L-K\}$. Then ${\tilde{G}}_{12}$ is defined as ${\tilde{G}}_{12}=(V_{1}\cup V_{2},{Ê}_{1}^{0}\cup {Ê}_{2}^{0})$, where ${Ê}_{1}^{0}=\{(v,w)\in {Ê}_{1}:w\leq m^{*}\}$ and ${Ê}_{2}^{0}=\{(v,w)\in {Ê}_{2}:w>m^{*}\}$.

The rationale for this is that marginal models may include spurious associations on the boundaries. For example, let $G_{1}$ and $G_{2}$ be the subgraphs of G induced by *V*_1_ and *V*_2_, respectively. Then the marginal distribution of *V*_2_ will not in general be $G_{2}$-Markov, but it will be $G_{2}^{*}$-Markov for a graph $G_{2}^{*}$ derived from $G_{2}$ by completing the boundary of each connected component of $G_{V\setminus V_{2}}$ in G[28]. So ${\hat{G}}_{2}$ will tend to contain edges not in $G_{2}$. To estimate the boundary of *V*_1_∖*V*_2_ in G, ${\hat{G}}_{1}$ is used: the boundary is estimated to be contained in the set $L-K+1,\ldots ,m^{*}$. Hence by only including edges $(v,w)\in {Ê}_{2}$ with *w*>*m*^∗^ edges in this boundary are omitted. Similarly, the boundary of *V*_2_∖*V*_1_ in $G_{1}$ may contain spurious associations, so ${Ê}_{1}^{0}$ may contain unnecessary edges. To avoid this the overlap length *K* is chosen to be sufficiently large so that *m*^∗^< min{*v*:∃ (*v*,*w*)∈*E*_2_ with *w*≥*L*+1}. If the maximum width was known in advance this inequality could be ensured by setting *K* to be twice the maximum width, but so large a value appears to be unnecessary in practice.

The algorithm proceeds in the obvious fashion combining ${\tilde{G}}_{12}$ with ${\hat{G}}_{3}$ and so on. Since the construction may result in chordless cycles it is necessary to triangulate the resulting graph $\tilde{G}$. This can be done using the maximum cardinality search algorithm [29] which can be implemented in linear time.

Assuming that the time for deriving the estimates ${\hat{G}}_{i}$ for fixed *L* is bounded, the algorithm described here is by construction linear in *p*. But the proportionality constant can be expected to be depend on various factors, such as the density of the minimum AIC/BIC model.

### Implementation

The methods are implemented in a set of R functions which are provided as Additional files 1 and 2 to the paper. The selection algorithms build on the forward search algorithm implemented in the stepw function in the gRapHD package [30]. Currently this function requires that there are no missing data. The backward search algorithm was developed by the author in the course of preparing this paper, and has subsequently been implemented in the gRapHD package. The functions to perform the selection algorithms, and others to work with and display the graphs, build on the following packages: igraph [31], graph [32], Rgraphviz [26] and gRapHD. The triangular heatmaps were produced using the LDheatmap package [33]. Simulation from a fitted decomposable model was carried out using the gRain package [34]. The package rJPSGCS provides an interface between R and the FitGMLD program which was used to perform the algorithm of [15].

## Results

To illustrate the methods described above, they were applied to SNP data obtained from three commercial pig breeds. In all 4239, 1979 and 2123 pigs of the Duroc, Landrace and Yorkshire breeds were genotyped using the Illumina Porcine SNP60 BeadChip [35]. After preprocessing on the basis of call rate, minimal allele frequency and other quality criteria, missing values were imputed using BEAGLE [22]. Using the methods described above, decomposable graphical models were selected for each chromosome and breed, using data at the genotype level. The BIC penalizing constant *α*= log(*N*) was used throughout.

**Figure 1:**
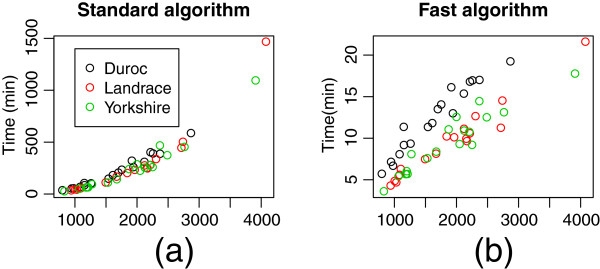
**Running times of the two algorithms.** The running times (in minutes) of the forward step of the standard and fast algorithms for the different chromosomes and breeds are shown in **(a)** and **(b)**, respectively.

Figure 1 compares the running times of the two algorithms, and confirms that the fast algorithm is approximately linear in the number of SNPs. Table 1 gives more detailed information. It is seen that the estimate $\tilde{G}$ is indeed a good approximation to $\hat{G}$, and that the algorithm is considerably faster than the standard greedy algorithm.

**Table 1:** The performance of the two algorithms

**Breed**	**chr**	***N***	***p***	***|E***_***s***_***|***	**under**	**over**	***|E***_***sp***_***|***	**under**	**over**	***t***_***s***_	***t***_***f***_	***t***_***sp***_	***t***_***fp***_	**width**
duroc	1	4249	2863	4762	0	0	4340	0	0	35084	1153	37	37	15
duroc	2	4249	2113	3676	0	3	3339	2	2	18450	920	26	27	10
duroc	3	4249	1535	2873	1	13	2643	4	15	8695	679	20	20	8
duroc	4	4249	2211	4392	0	0	4012	0	0	24005	1007	35	36	11
duroc	5	4249	1255	2357	7	2	2166	8	2	6030	559	16	16	10
duroc	6	4249	1937	3358	0	0	3069	0	0	15396	778	24	24	12
duroc	7	4249	1911	3978	0	3	3621	0	2	19112	966	36	36	12
duroc	8	4249	1694	3136	3	3	2871	3	3	12193	808	24	24	12
duroc	9	4249	1749	3399	3	14	3087	5	17	13904	841	26	27	14
duroc	10	4249	1078	2178	0	0	1980	0	0	3991	482	15	15	12
duroc	11	4249	1145	2493	0	2	2294	0	3	6206	680	19	19	16
duroc	12	4249	973	1857	0	0	1689	0	0	3138	399	13	13	10
duroc	13	4249	2365	3998	0	7	3634	0	2	23112	1019	29	29	9
duroc	14	4249	2252	4037	0	3	3731	0	2	23291	1016	29	29	14
duroc	15	4249	1605	2977	0	0	2717	0	0	10757	707	22	22	11
duroc	16	4249	1148	2249	0	0	2032	0	0	4936	549	16	16	10
duroc	17	4249	941	1856	1	3	1703	1	3	3034	427	12	12	10
duroc	18	4249	792	1402	0	0	1262	0	0	2032	341	9	10	9
landrace	1	1979	4071	7679	0	5	7020	0	4	87941	1295	63	64	12
landrace	2	1979	2153	4058	0	0	3690	0	0	14868	595	28	29	12
landrace	3	1979	1957	4036	1	5	3664	4	3	13841	605	30	31	17
landrace	4	1979	2210	4498	1	1	4111	3	2	16629	634	33	33	12
landrace	5	1979	1489	3368	0	7	3090	1	4	6511	447	24	25	16
landrace	6	1979	2163	4113	0	0	3753	0	0	14664	577	28	28	12
landrace	7	1979	2116	4634	0	0	4238	0	0	16385	666	36	37	13
landrace	8	1979	1836	3989	2	4	3682	4	4	11989	611	28	29	14
landrace	9	1979	2301	4897	0	4	4415	0	5	20077	758	40	40	13
landrace	10	1979	1077	2285	3	11	2074	9	9	2997	334	16	16	14
landrace	11	1979	999	2150	0	0	1954	0	0	2492	289	15	15	11
landrace	12	1979	1098	2573	0	0	2332	0	0	3420	376	21	21	11
landrace	13	1979	2734	5295	0	7	4868	3	6	30046	870	39	40	12
landrace	14	1979	2709	4819	1	4	4454	5	3	26602	673	33	33	17
landrace	15	1979	1666	3507	8	13	3202	10	12	9888	486	25	25	15
landrace	16	1979	1181	2403	0	0	2179	0	0	3747	339	15	16	10
landrace	17	1979	1025	2227	1	3	2017	3	3	2390	279	15	15	11
landrace	18	1979	934	1890	0	2	1721	3	3	1981	256	12	12	9
yorkshire	1	2123	3904	6700	1	3	6112	1	1	65538	1065	51	52	11
yorkshire	2	2123	2202	4311	1	8	3968	3	9	16548	644	32	33	11
yorkshire	3	2123	1994	4440	0	9	4016	1	10	17020	751	39	39	13
yorkshire	4	2123	2246	4359	3	3	3979	4	3	15429	550	32	32	12
yorkshire	5	2123	1525	3145	0	0	2864	0	0	6471	454	22	22	16
yorkshire	6	2123	2045	3876	2	2	3513	2	2	13313	556	27	27	11
yorkshire	7	2123	2363	5342	0	11	4894	4	10	27929	866	44	46	14
yorkshire	8	2123	1868	3887	1	5	3593	1	3	14254	662	27	28	11
yorkshire	9	2123	2119	4174	0	3	3757	1	5	15108	653	31	31	11
yorkshire	10	2123	1193	2518	0	0	2268	0	0	3757	360	19	19	12
yorkshire	11	2123	1269	2851	0	0	2633	0	0	5501	483	20	20	12
yorkshire	12	2123	1061	2287	0	0	2068	0	0	2885	324	17	17	10
yorkshire	13	2123	2759	4935	0	0	4544	0	0	27100	785	35	35	13
yorkshire	14	2123	2482	4732	0	6	4353	1	4	22286	749	34	33	11
yorkshire	15	2123	1669	3321	0	4	3030	2	5	8385	501	24	24	12
yorkshire	16	2123	1207	2575	1	5	2345	2	5	3734	339	17	17	10
yorkshire	17	2123	1176	2446	2	7	2223	3	8	3621	339	16	17	14
yorkshire	18	2123	825	1618	2	6	1445	6	6	1434	215	10	11	9

The table shows the performance of the algorithms applied to the pig data. *N* and *p* denote the numbers of observations in the data and number of SNPs available after filtering. The edge sets found in steps 1 and 2 of the standard algorithm are denoted *E*_*s*_ and *E*_*sp*_, respectively: for the fast algorithm they are denoted *E*_*f*_ and *E*_*fp*_. The numbers of undershoots and overshoots, i.e. |*E*_*s*_∖*E*_*f*_| and |*E*_*f*_∖*E*_*s*_| for step 1, and |*E*_*sp*_∖*E*_*fp*_| and |*E*_*fp*_∖*E*_*sp*_| for step 2, are also shown. The corresponding running times in seconds are denoted *t*_*s*_, *t*_*f*_, *t*_*sp*_ and *t*_*fp*_, respectively. The backward step is much faster than the forward step in both cases (*t*_*sp*_ and *t*_*fp*_). Overall, the fast algorithm is 23.4 times faster than the standard algorithm, and its inaccuracy, assessed as $\sum (|E_{s}\setminus E_{f}|+|E_{f}\setminus E_{s}|)/\sum (|E_{s}|)$, is 0.0012. The last column shows the maximum width of $G=(V,E_{s})$. The computations were run under Redhat Fedora 10 Linux on a Intel i7 four-core 2.93GHz machine with 48 GB RAM.

**Figure 2:**
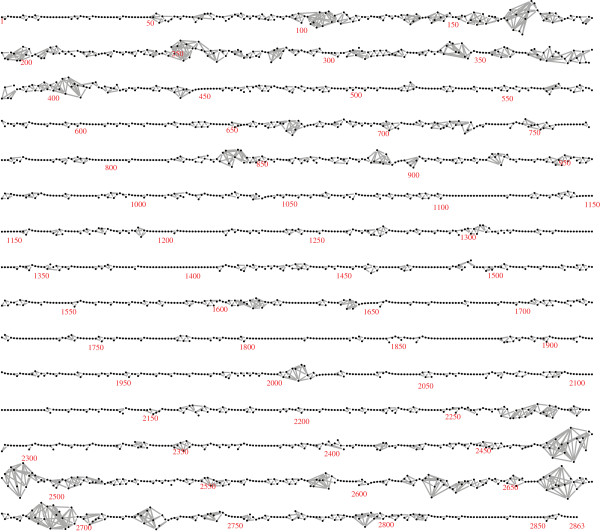
**The LD graph for Duroc chromosome 1.** The estimated LD graph for chromosome, constructed using the unphased genotypes, is shown.

Figure 2 shows the LD graph for one chromosome (Duroc chromosome 1): the same graph is obtained with both algorithms. It is a long thin graph with 2863 nodes and 4340 edges. It has 32 connected components, of which 20 are isolated vertices — suggesting SNP positioning errors — and four are long intervals with 997, 569, 786 and 452 SNPs. Curiously, the subgraph induced by the last 35 SNPs contains 7 connected components, which suggests positioning errors in this region. The most striking feature of the graph is that for about 80% of the chromosome, a simple serial or near-serial association structure is found, but for the remaining 20% more complex patterns of LD are observed. Similar results are found for all chromosomes and breeds.

**Figure 3:**
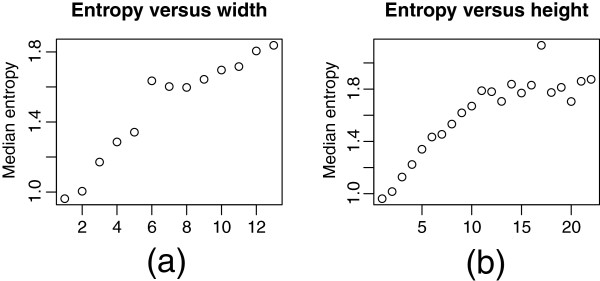
**Entropy versus height and width for Duroc chromosome 1.** In **(a)** a plot of median entropy, a measure of haplotype diversity, versus width for Duroc chromosome 1 is shown. In **(b)**, a corresponding plot of median entropy versus height is shown. The entropy was calculated for each interval of 7 adjacent SNPs using the phased data.

**Figure 4:**
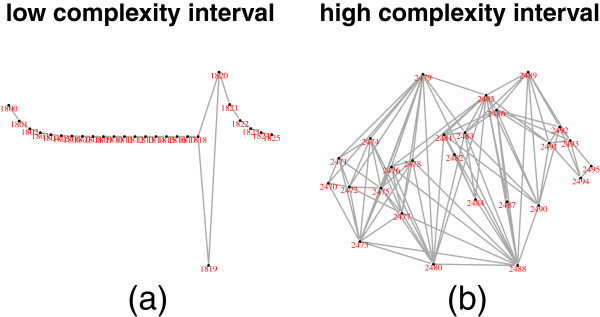
**The subgraphs of typical low and high complexity intervals.** The subgraphs of a typical low complexity interval (SNPs 1800-1825) and high complexity interval (SNPs numbered 2470-2495) are shown in **(a)** and **(b)**, respectively.

**Figure 5:**
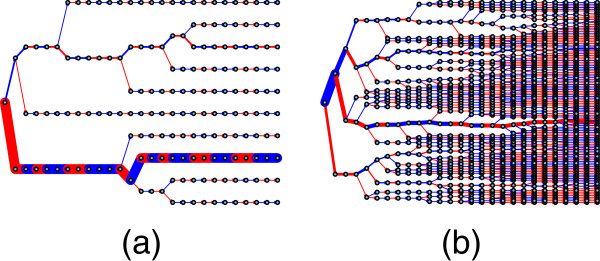
**The sample tree for the low and high complexity intervals.** Sample trees for the low and high complexity intervals are shown in **(a)** and **(b)**, respectively. The trees are based on the phased haplotype data. The root node is drawn on the left. Edge widths are proportional to the numbers of observations passing through the edges. The colour of the edges signifies the allele: red is 1 and blue 2. It is seen that the interval shown in **(a)** has low haplotype diversity, dominated by a single common haplotype, whereas that shown in **(b)** has high haplotype diversity, with one or two relatively common haplotypes.

**Figure 6:**
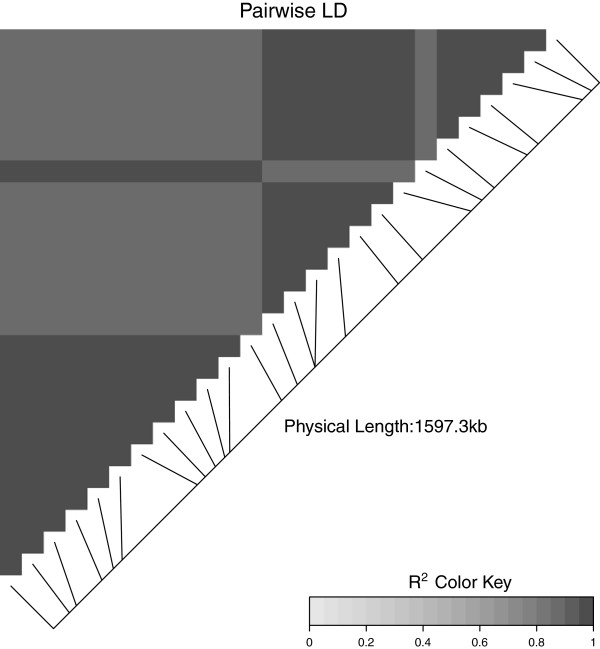
**A triangular heatmap for the low complexity interval.** A heatmap of the pairwise LD values in the low complexity interval is shown. All associations are high.

**Figure 7:**
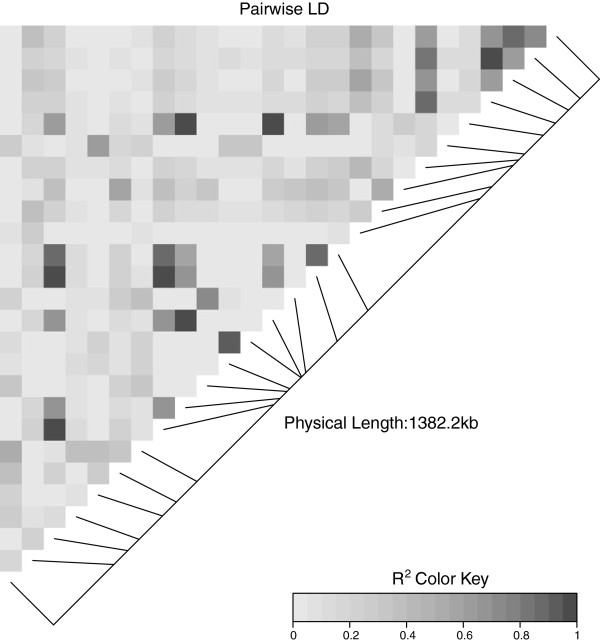
**A triangular heatmap for the high complexity interval.** A heatmap of the pairwise LD values in the high complexity interval is shown. The LD values form a mosaic of mostly low associations.

Genomic intervals with simple association structure tend to be associated with low haplotype diversity, and intervals with complex structure with high diversity (Figure 3). To further compare the low and high complexity regions, two representative intervals of the same length were selected: SNPs numbered 1800-1825 (low complexity), and SNPs numbered 2470-2495 (high complexity). These have sample entropies of 0.79 and 4.05, respectively. Their subgraphs are shown in Figure 4, and their sample trees in Figure 5. These show the low diversity of the low complexity interval, and the relatively high diversity of the high complexity interval. Corresponding triangular heatmaps are shown in Figures 6 and 7. The low complexity interval has high LD whereas the high complexity interval shows a more mosaic structure.

Thus low complexity regions tend to consist of series of haplotype blocks with high LD and low haplotype complexity, and may be dominated by a few common haplotypes. In contrast, regions of high complexity tend to have high haplotype diversity and little or no haplotype block structure. It must be stressed that the SNPs in such regions are not generally in linkage equilibrium: on the contrary, complex patterns of association, not marginal independences, are observed.

**Figure 8:**
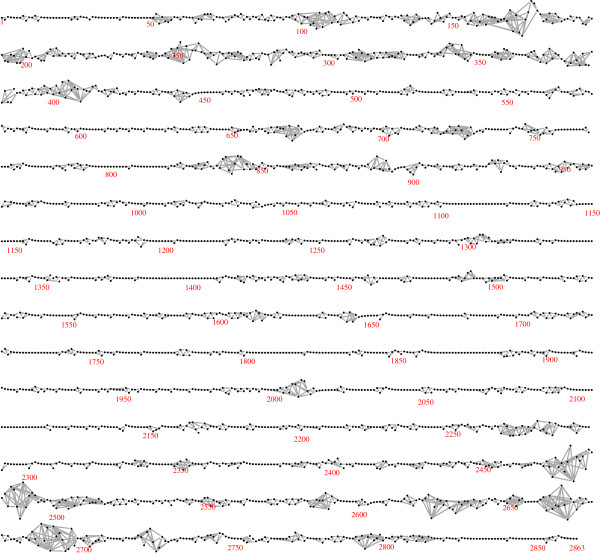
**The LD graph for Duroc chromosome 1 based on phased data.** The estimated LD graph for Duroc chromosome 1, constructed using the phased haplotypes, is shown.

Figure 2 and Table 1 show the results of applying the algorithms to unphased, genotype data, but as mentioned above they may also be applied to phased haplotype-level data. This implicitly regards the inferred haplotypes as a random sample of size 2*N* from an underlying population of haplotypes. Analyses based on inferred haplotypes may be subject to a loss of efficiency [36]. For the current data phase imputation was carried out using the BEAGLE software [22]: applying the fast algorithm to the phased data for Duroc chromosome 1 resulted in the graph shown in Figure 8. This is slightly denser than the graph in Figure 2, with 375 more edges. This may be ascribed to the reduced model dimension due to the use of binary rather than trichotomous variables, which leads to a weaker penalization of complex models in (1). Haplotype- and genotype-level graphical models are related but in general distinct: it has been shown that they are identical only when the haplotype-level graph is acyclic [37].

**Figure 9:**
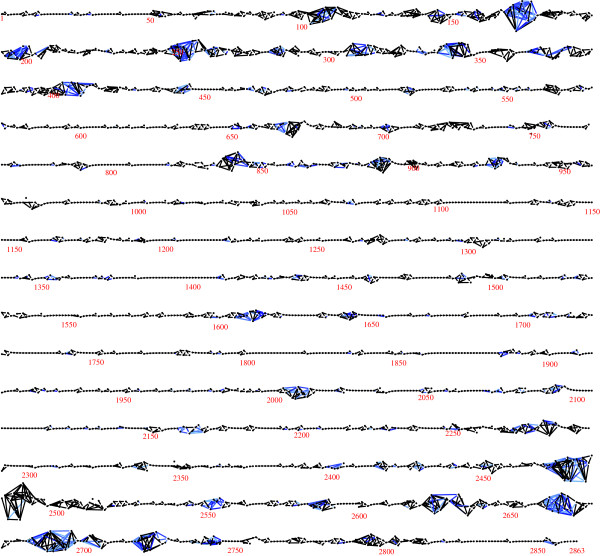
**The union of LD graphs for Duroc chromosome 1 derived from ten simulated data sets.** The figure represents the results of applying the fast algorithm to ten data sets simulated under the LD graph shown in Figure 1. The union of the ten selected graphs is shown. Edges present in all ten graphs are black, and the colour of the others is interpolated between light blue (those found once) and blue (those found nine times).

To assess the accuracy of the algorithm, twenty simulated data sets each with the same number of observations as the data sets analyzed above (*N*=4239) were constructed. The first ten were generated by taking *N* random samples from the skeleton, that is, the graphical model with edges between physically adjacent SNPs only, fitted to the Duroc chromosome 1 genotype data. The second ten were generated by taking *N* random samples from the model shown in Figure 2, fitted to the same data. The results of applying the fast algorithm to each data set are summarized in Table 2. For the skeleton, the edgewise false negative rate was 0.029 and the edgewise false inclusion rate was 0.00024. For the model in Figure 2 the corresponding rates were 0.031 and 0.019. To visually represent the latter results, Figure 9 shows a graph $G=(V,\cup_{j}E_{j})$, in which the edge colours represent the frequency that the edge was found in the 10 models. The graph suggests that model uncertainty is primarily restricted to the genomic intervals with complex dependence structure.

**Table 2:** Results from simulations

**Skeleton**		**LD graph**	
**Undershoot**	**Overshoot**	**Undershoot**	**Overshoot**
81	0	117	73
84	0	146	82
82	0	138	81
84	0	122	94
83	0	161	84
76	1	141	77
88	2	127	70
81	0	133	92
79	3	138	79
85	1	133	90

The table summarizes the inaccuracy of the fast selection algorithm applied to data sets simulated under two models for Duroc chromosome 1: the skeleton, and the more complex LD graph shown in Figure 2. The columns labelled undershoot and overshoot show |*E*∖*E*_*j*_| and |*E*_*j*_∖*E*|, where *E* and *E*_*j*_ are the edge sets of the true model and the model found by the algorithm, respectively. For the data sets simulated under the skeleton, the edgewise false negative rate $\frac{1}{10|E|}\sum_{j}|E\setminus E_{j}|$ is 0.029 and the false positive rate $\frac{1}{10|E|}\sum_{j}|E_{j}\setminus E|$ is 0.00024. Under the more complex LD graph, the corresponding rates are 0.031 and 0.019.

**Figure 10:**
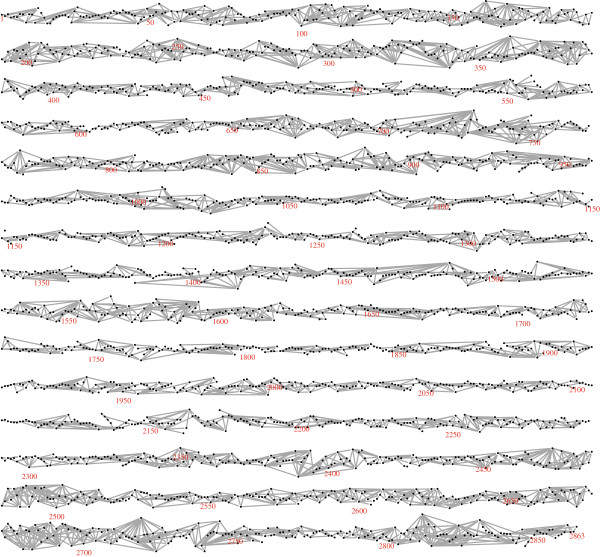
**Haplotype-level graph found using the algorithm of [**[15]**].** The figure shows the result of applying the algorithm of [15] to the same data set as used with Figure 2. The algorithm estimates a haplotype-level graph for the data.

Finally, for comparison purposes the algorithm of [15] was applied to the Duroc chromosome 1 genotype data. This algorithm automatically imputes phase and missing marker data, cycling between imputation and model selection as sketched in the background section. The selected graph is shown in Figure 10. Like the graphs shown in Figures 2 and 8, it is long and stringy, but rather more dense. This greater density may be explained by the use in [15] of a Metropolis acceptance rule that is based on the penalized likelihood (1), but with a smaller penalizing constant *α*=(1/8) log(*N*). A detailed comparison of the two algorithms would be valuable but is not attempted here.

## Discussion

This paper has introduced an efficient, linear-time forward-backward algorithm to estimate chromosome-wide probabilistic graphical models of fine-scale linkage disequilibrium, and has described a convenient way to display these models. In illustration, the methods have been applied to data obtained from three commercial breeds of pigs using the Illumina Porcine SNP60 BeadChip.

The forward part of the algorithm proceeds by combining a series of overlapping marginal decomposable models of dimension *L* and overlap length *K*. This implicitly assumes that the maximum width of the true graph is at most *K*. The resulting model is then triangulated and backward selection performed. The search space closely resembles that of [15], which samples from decomposable models with a given maximum width, using sliding windows. The difference in approaches lies primarily in the search method: here greedy search to optimize a penalized likelihood criterion is used, whereas in [15] MCMC sampling methods are applied.

The R function used for greedy forward search currently requires that the input data contain no missing values, so it was necessary to impute missing values prior to performing the algorithm, and the BEAGLE software [22] was used for this. This raises the possibility of circularity, or more precisely, that the model selection is influenced by constraints or assumptions implicit in the models used by BEAGLE. But given BEAGLE’s high imputation accuracy with such data it seems unlikely that this plays an important role here.

The algorithm was found to have high accuracy when applied to simulated data sets of the dimensions considered here (*N*∼4000;*p*∼3000), with edgewise false positive and negative rates of around 3%. That it is good at reconstructing the model generating the data is reassuring. Needless to say, the algorithm does not necessarily identify the “true” model, which may not be in the search space. As noted previously, the approach captures short range associations, but not long range ones. Moreover, higher edgewise false negative rates may occur when observed data are used if there are infinitesimal departures from a model that are not detectable at the given sample size, as these are filtered out when data are simulated under a selected model.

A comparison of triangular heatmaps with LD graphs is instructive. The former are compact graphical representations of all pairwise marginal associations for a set of SNPs. They are particularly well-suited to identify LD blocks, which stand out as highlighted triangles. LD graphs supplement heatmaps by showing patterns of association but not their strength. Since they are parametric models they can be put to a number of quantitative uses as described in the background section. At one level, heatmaps and LD graphs convey similar information, since intervals with simple dependence structures tend to appear as series of LD blocks in the heatmap, whereas those with complex structures tend to occur in the inter-block regions and have a more mosaic appearance. But the graphs provide a more incisive characterisation of genetic variation, building on the concept of conditional rather than marginal dependence. In this regard, it may be helpful to regard conditional independence statements as expressing the notion of *irrelevance*, in the sense that *A*⊥ ⊥*B* | *C* implies that if we know *C*, information about *A* is irrelevant for knowledge of *B*. Thus the graphs say something about connections between specific SNPs, for example about which SNPs are required to predict a specific SNP.

The graphs may also be useful in fine mapping. Genome-wide association studies seek to find the genetic basis of complex traits, typically using single locus methods - that is, by identifying SNPs with strong marginal association with the complex trait. Due to LD, many SNPs in a genomic region may exhibit strong association with the trait, making it hard to identify the causal loci. A way to address this is to assess the effect of a putative causal SNP on the complex trait in a linear model that also includes terms for the two flanking SNPs, in order to adjust for confounding with nearby effects due to LD. This implicitly assumes a simple serial dependence structure between the SNPs, and when the dependence structure is more complex such adjustment might be insufficient, leading to false positives. This may be prevented by including terms for the neighbours of the putative causal SNP, not just for the flanking SNPs. Any SNP is separated from the remaining SNPs by its neighbours, so by the global Markov property it is independent of the remaining SNPs given its neighbours. A similar method has been proposed based on Bayesian networks [38].

Like graphical models, the APFA models that underlie BEAGLE may be represented as graphs that encode a set of conditional independence relations, but in a very different way. In the present context, for example, nodes in APFA graphs represent haplotype clusters rather than SNPs. Where the model classes intersect, APFA graphs are much more complex than the corresponding dependence graphs, and so less amenable to visualization.

A striking feature of the LD graphs for the pig data was that for roughly 80% of the genome, simple serial or near-serial LD patterns were found, but for the remaining 20%, more complex patterns were observed. The regions with the simple serial structure tend to have low haplotype diversity, which is to be expected in livestock breeds with small effective population sizes [39]. Perhaps more unexpected is that roughly 20% of the porcine genome exhibits complex LD patterns, forming islands of relatively high genetic diversity. This information may be useful in an animal breeding context, to identify regions with high genetic variation. It will also be interesting to compare graphs obtained using different SNP densities in a given breed or species to examine whether and how their topologies change with varying marker densities.

## Conclusions

The proposed algorithm is efficient and makes it feasible to estimate and visualize chromosome-wide LD models on a routine basis.

## Competing interests

The author declares he has no competing interests.

## Supplementary Material

Additional file 1 **LDgraph.** The R functions implementing the methods of the paper. Click here for file

Additional file 2 **exampleScript.** R code illustrating how the R functions may be used. Click here for file
